# *In Situ* Sampling of Relative Dust Devil Particle Loads and Their Vertical Grain Size Distributions

**DOI:** 10.1089/ast.2016.1544

**Published:** 2018-10-10

**Authors:** Jan Raack, Dennis Reiss, Matthew R. Balme, Kamal Taj-Eddine, Gian Gabriele Ori

**Affiliations:** ^1^ School of Physical Science, Faculty of Science, Technology, Engineering & Mathematics (STEM), The Open University, Milton Keynes, UK.; ^2^ Institut für Planetologie, Westfälische Wilhelms-Universität, Münster, Germany.; ^3^ Géologie et Géoinformatique, Faculté des Sciences Semlalia, Université Cadi Ayyad, Marrakech, Morocco.; ^4^ Ibn Battuta Centre, Faculté des Sciences Semlalia, Université Cadi Ayyad, Marrakech, Morocco.; ^5^ International Research School of Planetary Sciences, Università “G. D'Annunzio”, Pescara, Italy.

**Keywords:** Mars, Dust devils, Planetary science, Desert soils, Atmosphere, Grain sizes

## Abstract

During a field campaign in the Sahara Desert in southern Morocco, spring 2012, we sampled the vertical grain size distribution of two active dust devils that exhibited different dimensions and intensities. With these *in situ* samples of grains in the vortices, it was possible to derive detailed vertical grain size distributions and measurements of the lifted relative particle load. Measurements of the two dust devils show that the majority of all lifted particles were only lifted within the first meter (∼46.5% and ∼61% of all particles; ∼76.5 wt % and ∼89 wt % of the relative particle load). Furthermore, ∼69% and ∼82% of all lifted sand grains occurred in the first meter of the dust devils, indicating the occurrence of “sand skirts.” Both sampled dust devils were relatively small (∼15 m and ∼4–5 m in diameter) compared to dust devils in surrounding regions; nevertheless, measurements show that ∼58.5% to 73.5% of all lifted particles were small enough to go into suspension (<31 μm, depending on the used grain size classification). This relatively high amount represents only ∼0.05 to 0.15 wt % of the lifted particle load. Larger dust devils probably entrain larger amounts of fine-grained material into the atmosphere, which can have an influence on the climate. Furthermore, our results indicate that the composition of the surface, on which the dust devils evolved, also had an influence on the particle load composition of the dust devil vortices. The internal particle load structure of both sampled dust devils was comparable related to their vertical grain size distribution and relative particle load, although both dust devils differed in their dimensions and intensities. A general trend of decreasing grain sizes with height was also detected.

## 1. Introduction

Dust devils are small vertical convective vortices that occur on Earth and Mars (*e.g.,* Thomas and Gierasch, 1985; Balme and Greeley, 2006). On Earth, dust devils are most common in semiarid to arid regions during spring and summer (*e.g.,* Ives, 1947), where they are formed by insolation under clear skies (Balme and Greeley, 2006). Dust devils consist of a low-pressure region in the interior that is surrounded by tangential winds and updrafts (*e.g.,* Sinclair, 1973; Newman *et al.,* 2002). Entrained particle sizes (dust and sand) lifted by the dust devil make them visible (*e.g.,* Sinclair, 1969; Balme and Greeley, 2006), while vortices without lifted particles can remain invisible and difficult to detect (*e.g.,* Hess and Spillane, 1990).

Dust devils are erosional features (Balme and Greeley, 2006) that sometimes leave dark (*e.g.,* Rossi and Marinangeli, 2004; Reiss *et al.,* 2010, 2013) or bright (Reiss *et al.,* 2011a) tracks on the surface. Mineral aerosols entrained into the atmosphere by dust devils have an influence on the terrestrial climate (*e.g.,* Gillette and Sinclair, 1990; Balme and Greeley, 2006) and are important for human health, weather, and biogeochemistry (Mahowald *et al.,* 2014) in that they absorb the incident sunlight (Renno *et al.,* 2004). Lifted small mineral aerosols (particles smaller than ∼25 μm in diameter) can be entrained into the atmosphere where they can be transported in suspension over long distances on Earth (Gillette and Sinclair, 1990; Balme and Greeley, 2006). Larger particles, especially sand-sized particles, remain at lower heights without going into suspension and can build up the so-called “sand skirt” of a dust devil (*e.g.,* Balme and Greeley, 2006; Whelley and Greeley, 2008). This sand skirt represents a local redistribution of surface material and reinforces the erosional significance of dust devils.

On Mars, in contrast to Earth, dust devils often leave dark or bright filamentary tracks on the surface (*e.g.,* Veverka, 1976; Malin and Edgett, 2001; Greeley *et al.,* 2005; Cantor *et al.,* 2006), which confirms their erosional potential. Small mineral aerosols (dust) are mainly lifted into the atmosphere by near-surface wind stress and dust devils (*e.g.,* Newman *et al.,* 2002). Particles smaller than ∼20 μm in diameter can go into suspension and probably be transported across the whole planet (Newman *et al.,* 2002). Whelley and Greeley (2008) estimated that dust devils may lift approximately one-half of the annual dust lifted in nonglobal dust storm years, and Kahre *et al.* (2006) also calculated that dust devils can lift one-half of the global dust into the atmosphere. Furthermore, dust devils on Mars are substantially larger than those on Earth (*e.g.,* Renno *et al.,* 2004; Reiss *et al.,* 2011b) and therefore likely contribute to a higher dust input into the martian atmosphere than occurs on Earth. These results show that dust devils play a significant role as a source for dust in the martian atmosphere and its climatic influences (Renno *et al.,* 2004; Balme and Greeley, 2006; Haberle *et al.,* 2006).

Here, we report on *in situ* sampling of the dust load and grain size distribution in different sample heights of two dust devils in the Sahara Desert in southern Morocco during a field campaign in the spring of 2012 (Raack *et al.,* 2014). *In situ* measurements of lifted particles in dust devil vortices were conducted by Mattsson *et al.* (1993), Oke *et al.* (2007), and Metzger *et al.* (2011) on three different continents on Earth (Africa, Australia, and North America, respectively). Furthermore, Oke *et al.* (2007) presented vertical grain size distributions of their *in situ* samples, which was also part of this work. Our work differs from that of Oke *et al.* (2007) in that (1) our sample height was significantly higher, (2) the sample intervals were larger, (3) the analyzed particle sizes were both smaller and larger in diameter, and (4) the grain size distribution was more detailed.

In the present study, we present detailed insight into the vertical grain size structure of two dust devils, which is crucial to our understanding of the general composition of dust devil vortices and advancement in future dust devil modeling. Our results indicate which particle sizes can be eroded and redistributed from the surface to build up a sand skirt. Also, our measurements allow for determination of the fraction of lifted particles that can reach suspension and those that will fall back to the surface. This is important for understanding the general input of mineral aerosols of dust devils into the atmosphere.

## 2. Study Area

**FIG. 1.**
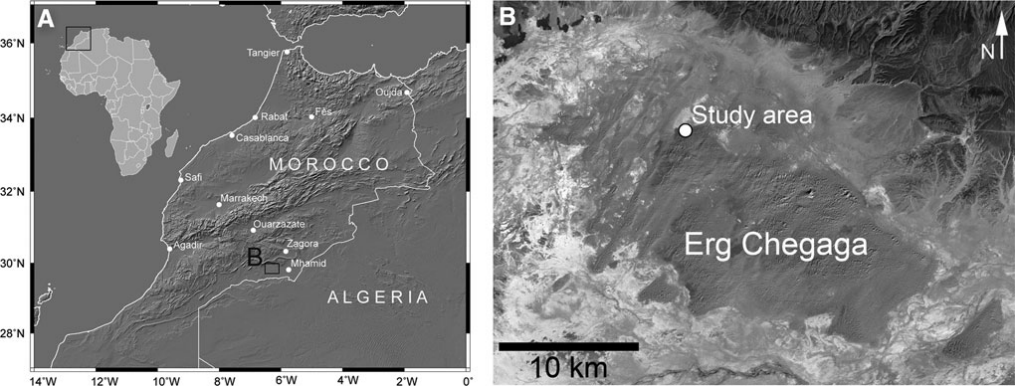
(**A**) Context of the study area. Black rectangle outlines image B [shaded relief map from SRTM4 data (Jarvis *et al.,* 2008)]. (**B**) Erg Chegaga and location of the study area (Landsat 7 RGB image).

**FIG. 2.**
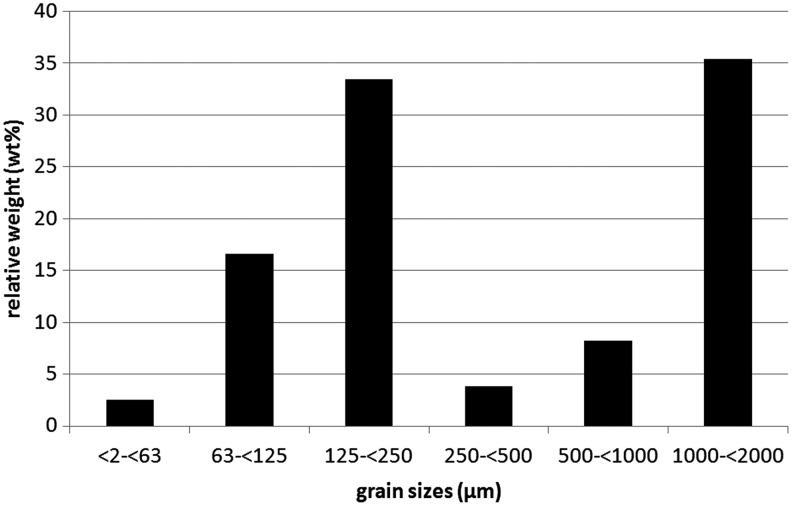
Grain size distribution of the surface materials within the study region [relative weight (wt %) vs. grain sizes (μm)]. Classification according to Udden (1914).

*In situ* sampling was conducted for two different dust devils on 22 April 2012 in a small study area on the northwestern rim of the Erg Chegaga dune field in southern Morocco (Fig. 1). The study area (29°53'8"N, 6°19'6"W) is approximately 60 km to the west of the small village M'hamid. The plain of the study area is characterized by a sandy surface with ripples. Ripple heights are between ∼5 and 7 cm and have wavelengths of about 50–75 cm. The grain size distribution of the surface is shown in Fig. 2. The main portions of the surface are very coarse sand (1000 to <2000 μm) with ∼35.4 wt % and fine sand (125 to <250 μm) with ∼33.7 wt % after the classification of Udden (1914). The grain size distribution of the ripples is bimodal, with the largest grains on top and finer-grained material beneath.

## 3. Data and Methods

For taking *in situ* samples of active dust devils, we used a 4 m high sampling boom made from aluminum pipe. The sampling areas are located on one side of the boom, where removable adhesive tape was mounted. This tape was covered prior to use to avoid contamination. Sampling involved holding the sampling boom upright and moving it into the path of the approaching dust devil. With this method, it was possible to take *in situ* samples from different heights within the dust devil. For our investigations, we took samples of two active dust devils to heights up to 2 and 4 m with a sampling interval of 0.25 and 0.5 m, respectively.

**FIG. 3.**
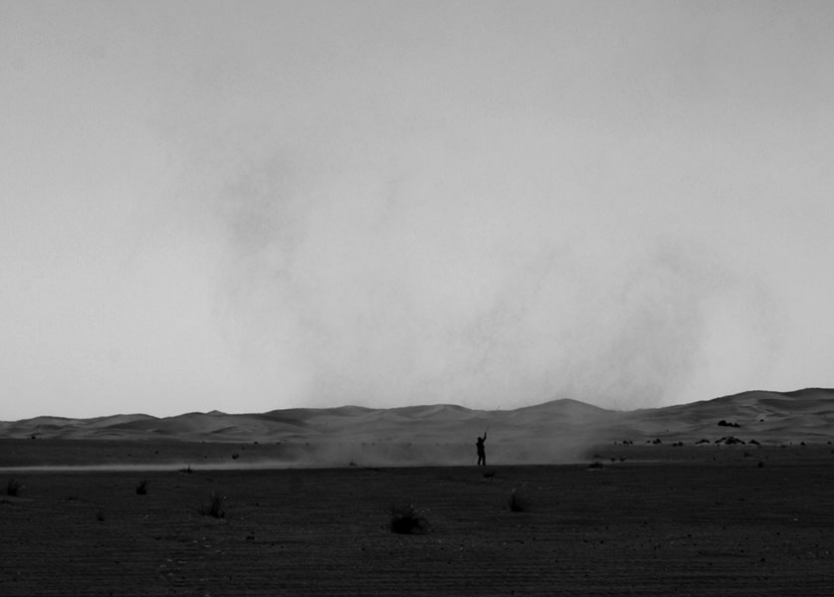
Image of DD #1 during *in situ* sampling. Note the author for scale.

For the sampling, the boom was positioned such that the sampling bisected the path of the oncoming dust devil (Fig. 3) and the dust devil passed the sampling device only once to avoid distortion of the results. After the passage of the dust devil, the sampling tape, which now had dust and sand grains adhered to it, was directly preserved on site by sticking the sample patches onto glass slides. This protected the samples from disruption and contamination and made further investigation with a microscope more practicable.

**FIG. 4.**
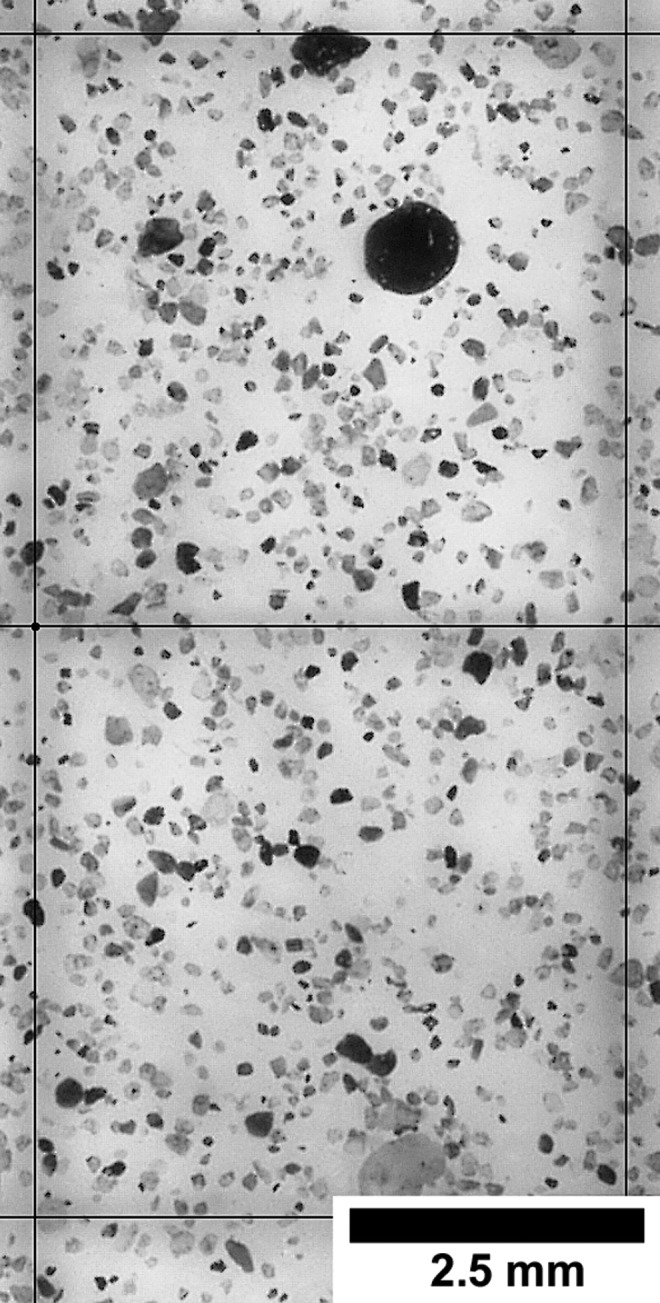
Example of a measured *in situ* sample (here: DD #1 at 0.5 m height). The maximum diameter of all grains within the two quadrangles (side lengths of 5 mm) was measured. Magnification is 20 × .

The samples recovered from the different heights were analyzed in the laboratory with an optical microscope. The samples were observed at a magnification of 200 × with the software package AnalySIS by Soft Imaging System (Fig. 4). With this software, the maximum diameter of all particles within a representative area of 0.5 cm^2^ was measured and recorded. Grain sizes were classified after the classification of Udden (1914) in three main groups (clay <2 to <4 μm, silt 4 to <63 μm, sand 63 to <2000 μm) and their individual finer grading (see also Table 2).

With the measured maximum diameter of each grain, it was possible to derive estimates of the percentage weight of the lifted particles (relative particle load). We calculated the volume of each measured grain under the assumption that it was perfectly rounded (spheres) and each grain had the same density. However, many of the measured grains were not perfect spheres and often had a more oval shape. Therefore, the presented values calculated with the measured maximum diameter are overestimated and give maximum volumes. After addition of all single grain volumes for each grain size classification, it was possible to derive the percentage of the weight (wt %) for each of these classification groups for any given hypothetical density.

Furthermore, for our detailed analyses, we calculated the mean and median values of the measured grain diameters for each aforementioned grain size classification group. The mean values are defined by the arithmetic mean, which shows the average of all measured grain diameters. The median defines the central measurement of each grain size classification group. This shows a more detailed picture of the grain size distribution compared to the mean value, which is often skewed to extremely large or small values.

## 4. Results

The analysis in this study is based on two different dust devils in the study area. One dust devil (DD #1) had a diameter of about 15 m (see Fig. 3). Here, the sample interval was 0.5 m for heights between 0.1 and 4 m. The second dust devil (DD #2) was weaker and smaller, with a diameter of approximately 4–5 m. The sample interval was 0.25 m for heights between 0.5 and 2 m.

**FIG. 5.**
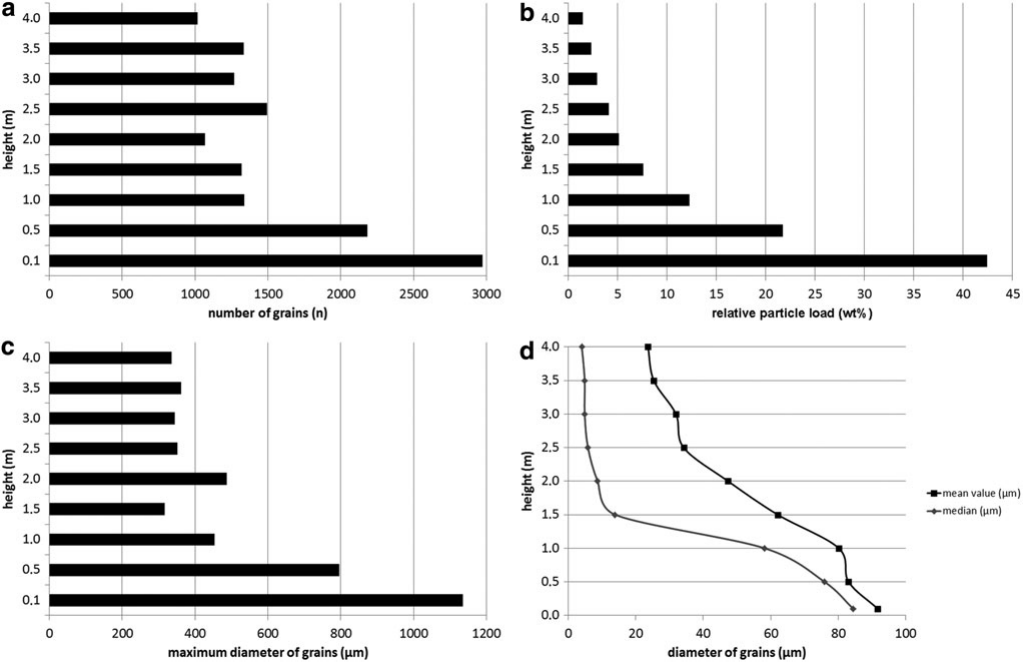
Measurements of dust devil #1 (DD #1). All measurements were taken within a representative 0.5 cm^2^ part of the sample area. (**a**) Number of measured grains vs. height. (**b**) Relative particle load (wt %) vs. height. (**c**) Maximum diameter of grains vs. height. (**d**) Mean value and median of the diameter of grains vs. height.

Figure 5a shows the number of grains (*n*) measured as a function of heights (m) in DD #1. The first sample height of 0.1 m was chosen to avoid disruption of the samples, which might have occurred in the first 10 cm during the deployment of the sampling boom onto the ground. The greatest number of particles (∼36.8% of the total) was sampled within the first 0.5 m. There was little variation in the number of particles detected at heights between 1 and 4 m (*n* was between about ∼1000 and ∼1500). In Fig. 5b, the relative particle load in weight percent is shown as a function of height. This shows the calculated mass of particles recorded in each height bin as a fraction of the total calculated mass of detected particles. The result shows a nearly exponential decrease of lifted particle load with height. The majority of all particles (∼76 wt %) was lifted within the first meter (Fig. 5b). Figure 5c shows the maximum diameter of grains observed at each sample height. The largest particles (1135 μm at 0.1 m height and 797 μm at 0.5 m height) were only found in the lowest 0.5 m (Fig. 5c). Above 0.5 m, the largest grain diameters found at each height are between ∼300 and ∼500 μm (Fig. 5c). In Fig. 5d, the median and mean values of the observed grain diameters are presented. Both values continuously decrease with height. The mean observed grain diameter decreases relatively linearly with height between 0.1 m and about 2.5 m (falling from ∼92 μm to ∼34 μm) but falls off more slowly between 2.5 m (∼34 μm) and 4 m (∼24 μm). The shape of this plot differs from the median data, which shows a rapid decrease in median grain diameter in the first 1.5 m (from ∼84 to ∼14 μm) and a very slow decrease with height above 1.5 m (from ∼14 to 4 μm).

**FIG. 6.**
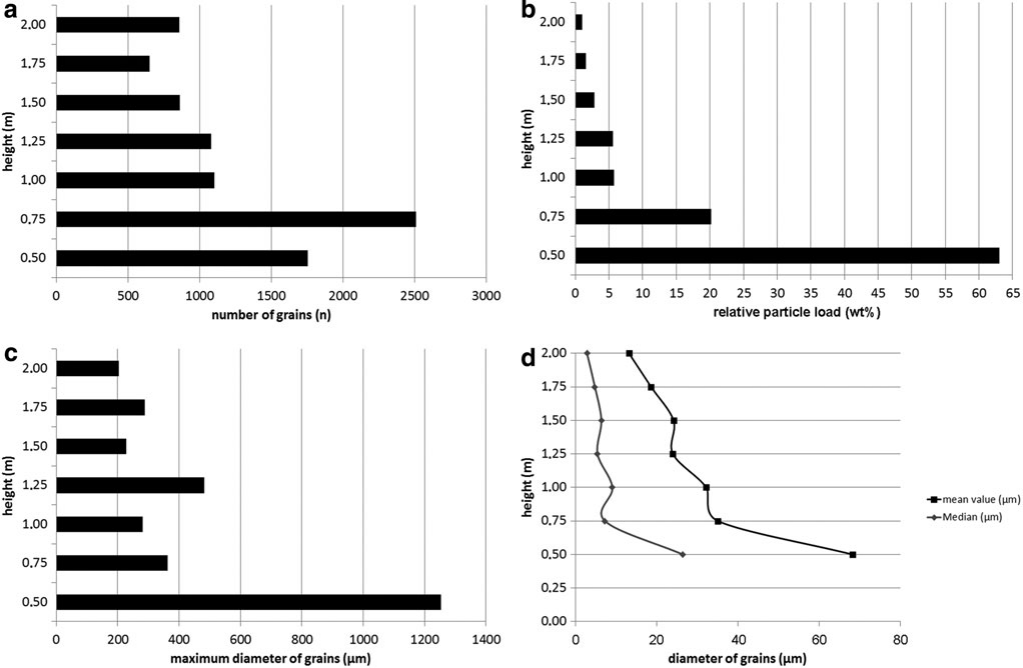
Measurements of dust devil #2 (DD #2). All measurements were taken within 0.5 cm^2^ of sample area. (**a**) Number of measured grains vs. height. (**b**) Relative particle load (wt %) vs. height. (**c**) Maximum diameter of grains vs. height. (**d**) Mean value and median of the diameter of grains vs. height.

Figure 6 shows similar plots to Fig. 5 but presented for DD #2. The number of grains (*n*) versus height (m) is presented in Fig. 6a, which shows that ∼48.4% of all particles were sampled within 0.75 m of the ground. Between heights from 0.75 to 2 m, there was again only slight variation in the number of observed grains. The mean number of grains observed at this height range was between ∼650 and ∼1100. The lifted relative particle load in weight percent is presented in Fig. 6b. Approximately 63 wt % of the total particle load was lifted within the first 0.5 m. Figure 6c shows that the largest grains (maximum diameter of ∼1254 μm) were lifted in the first 0.5 m, while grains between 0.75 and 2 m have maximum diameters of ∼200–500 μm. The mean value and median of the diameters of grains in micrometers (Fig. 6d) show nearly identical trends. After a relatively high value in both data sets (mean value: ∼68 μm; median: ∼26 μm) at 0.5 m height, both values decrease rapidly between height of 0.5 and 0.75 m to values of ∼35 μm for the mean and ∼7 μm for the median. Between 0.75 and 2 m, the mean value decreases from ∼35 to ∼13 μm, but the median is relatively constant, with values between ∼9 and ∼3 μm. All measurements are presented in Table 1.

**Table 1.** Absolute Values of Measurements of Both Dust Devils Presented in Figures 5 and 6

*DD #1*					
*Height (m)*	*Number of grains* (n)	*Maximum diameter of grains (μm)*	*Relative particle load (wt %)*	*Mean value of diameter (μm)*	*Median of diameter (μm)*
0.1	2971	1135.14	42.42	91.72	84.47
0.5	2182	796.54	21.75	83.09	75.95
1.0	1339	453.78	12.27	80.20	58.25
1.5	1321	316.03	7.59	62.12	13.90
2.0	1070	486.68	5.12	47.37	8.68
2.5	1493	352.17	4.13	34.20	5.83
3.0	1270	343.85	2.93	31.91	4.85
3.5	1333	362.12	2.31	25.34	4.85
4.0	1017	335.58	1.48	23.62	4.00

*DD #2*					
*Height (m)*	*Number of grains* (n)	*Maximum diameter of grains (μm)*	*Relative particle load (wt %)*	*Mean value of diameter (μm)*	*Median of diameter (μm)*
0.5	1755	1253.62	63.04	68.26	26.47
0.75	2511	363.08	20.19	35.08	7.26
1.0	1104	281.62	5.76	32.21	9.11
1.25	1082	482.45	5.63	24.03	5.45
1.5	861	227.29	2.80	24.17	6.39
1.75	652	288.58	1.58	18.65	4.81
2.0	858	204.24	1.00	13.21	2.86

**FIG. 7.**
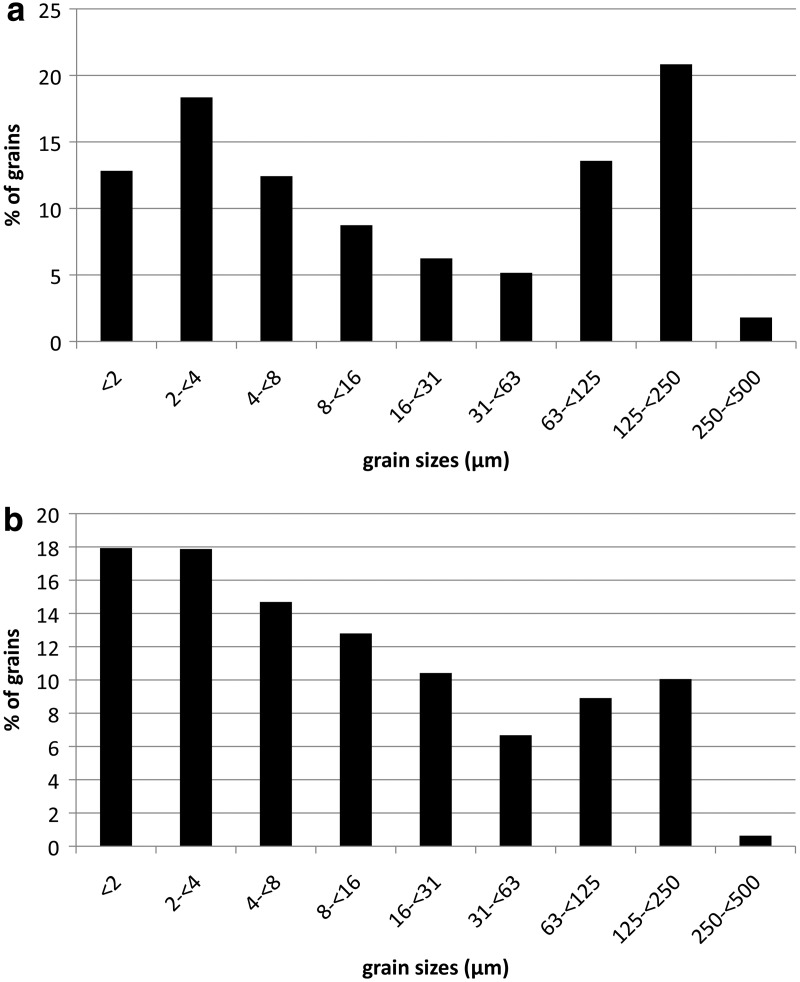
Grain size distribution for DD #1 (**a**) and DD #2 (**b**) after the classification of Udden (1914) from clay to medium sand.

In Fig. 7, the summarized grain size distributions, summed across all sample heights for both dust devils, are presented as a percentage of the total number of grains observed (after the classification of Udden, 1914). Sand grains with sizes above 500 μm were excluded in these diagrams because they were only very minor components (*e.g.,* only seven sand grains >500 μm in diameter were measured in samples from DD #1, and only two sand grains >500 μm in diameter were measured in samples from DD #2; see Table 2).

**Table 2.** Relative Abundance of Lifted Particles (See Fig. 7) and the Relative Lifted Particle Load for Both Dust Devils Grouped for Each Grain Size Classification

	*DD #1*		*DD #2*	
*Grain size*	*Lifted particles (%)*	*Lifted particle load (wt %)*	*Lifted particles (%)*	*Lifted particle load (wt %)*
Clay (<2 μm)	12.83	0.00003	17.93	0.00006
Clay (2 to <4 μm)	18.35	0.00025	17.87	0.00046
Very fine silt (4 to <8 μm)	12.42	0.0012	14.69	0.003
Fine silt (8 to <16 μm)	8.74	0.0074	12.80	0.02
Medium silt (16 to <31 μm)	6.24	0.04	10.42	0.12
Coarse silt (31 to <63 μm)	5.16	0.26	6.68	0.63
Very fine sand (63 to <125 μm)	13.58	7.19	8.91	8.52
Fine sand (125 to <250 μm)	20.83	54.85	10.05	48.78
Medium sand (250 to <500 μm)	1.80	27.10	0.63	18.13
Coarse sand (500 to <1000 μm)	0.04	5.27	0.01	2.04
Very coarse sand (1000 to <2000 μm)	0.01	5.27	0.01	21.76

The grain size distribution of DD #1 (Fig. 7a) shows a relatively high amount of clay, with the second highest value (∼18%) for clay in the bin for diameters between 2 and <4 μm. Silt-grade material has lower abundance and is constantly decreasing in abundance with grain size from ∼12.5% for very fine silt (4 to <8 μm) to ∼5% for coarse silt (31 to <63 μm). Fine sand (125 to <250 μm) provided the largest contribution with over 20% of the total number of grains being found in this bin. The lowest size fraction is that for medium sand (250 to <500 μm), which has an abundance of only ∼2%.

The grain size distribution for DD #2 is different from that of DD #1 in some regards, although the overall shape of the distribution is similar (Fig. 7b). The most abundant particles have clay-grade grain sizes (the two smallest particle size bins each account for about 18% of the total number of grains). Similar to DD #1, silt-grade materials constantly decrease in abundance with grain size from very fine silt (4 to <8 μm; ∼14.5% abundance) to coarse silt (31 to <63 μm; ∼6.5%), but silt in general is found in higher amounts than in DD #1. Also in contrast to DD #1 is the lower amount of sand: very fine sand (63 to <125 μm) and fine sand (125 to <250 μm) have abundances of only ∼9% and ∼10%, respectively. Medium sand is again the lowest fraction with only ∼0.5% of the total number of observed particles.

All the absolute values for Fig. 7 are provided in Table 2. Furthermore, the table contains values for the relative lifted particle load (in wt %) for each grain size classification. Fine sand comprises the largest portion of the total particle load in both dust devils (∼55 wt % for DD #1 and ∼49 wt % for DD #2), but small particles like clay and silt only contribute a very small amount to the total lifted particle load (∼0.3 wt % for DD #1 and ∼0.8 wt % for DD #2).

**FIG. 8.**
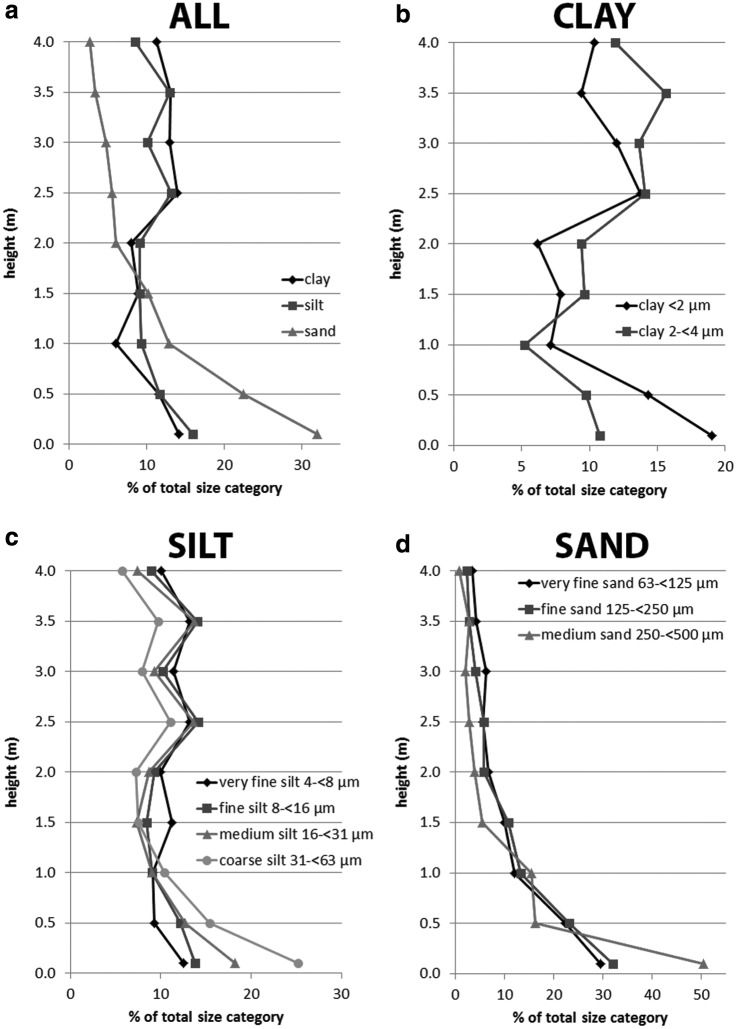
(**a**) Relative values of the total distribution of the different particle sizes (clay, silt, and sand) within the DD #1 vortex. (**b**–**d**) Relative values of the total distribution of clay (b), silt (c), and sand (d) within the DD #1 vortex.

**FIG. 9.**
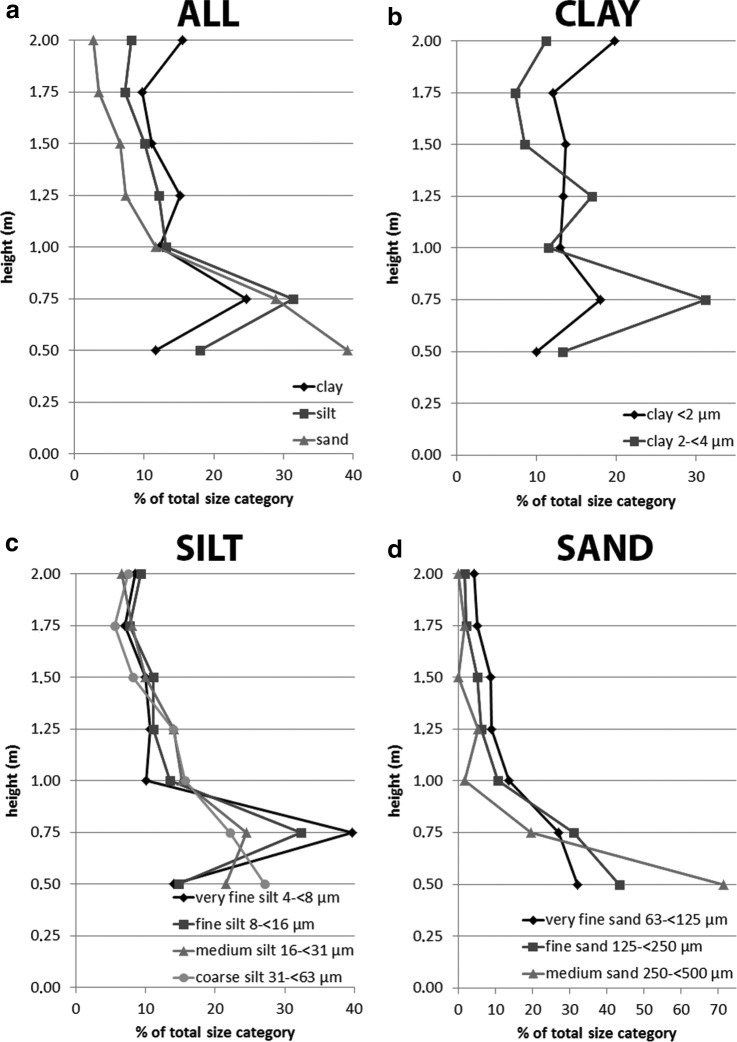
(**a**) Relative values of the total distribution of the different particle sizes (clay, silt, and sand) within the DD #2 vortex. (**b**–**d**) Relative values of the total distribution of clay (b), silt (c), and sand (d) within the DD #2 vortex.

More detailed views of the grain size distributions for every sample height of DD #1 and #2 are presented in Figs. 8 and 9. Figure 8a shows the amount of clay (<4 μm), silt (4 to <63 μm), and sand (63 to <500 μm) as a percentage of the total number of grains in that size category as a function of sample height for DD #1. Again, sand grains with sizes above 500 μm were excluded due to their low numbers. It can be seen clearly that clay and silt grains are distributed relatively evenly as a function of height but that sand grains show a strongly decreasing abundance with height. The largest fraction of sand grains are found in the lower parts of the dust devil, between 0.5 and 1.0 m (∼32% at 0.1 m, ∼22.5% at 0.5 m, and ∼13% at 1.0 m). Above 1.5 m height, sand grain abundance decreases constantly from ∼6% to ∼2.5%. Figure 8b shows the distribution of clay grains in detail, grouped into two ranges: particle sizes <2 μm and particle sizes between 2 and <4 μm. Here, the distribution is very irregular, and no clear trend is seen. The detailed distribution of silt grains, grouped into very fine silt (4 to <8 μm), fine silt (8 to <16 μm), medium silt (16 to <31 μm), and coarse silt (31 to <63 μm), is shown in Fig. 8c. Medium and coarse silt form a large part of the size range in the first 10 cm above the ground, but at heights above 1 m, the distribution of all the silt is generally relatively constant, with only minor variations between ∼7.5% and ∼14%. Only coarse silt has constantly lower amounts than other silt categories at heights above 1.5 m. In Fig. 8d, the detailed distribution of sand grains is presented and grouped into very fine sand (63 to <125 μm), fine sand (125 to <250 μm), and medium sand (250 to <500 μm). The general trend shows that the abundance of sand in all categories decreases with height. There are some variations in the first 1–1.5 m, but at greater heights, all grades of sand decrease to a very low abundance. Most sand grains were only lifted to within the first meter, especially medium sand, which shows values of ∼50% abundance at 0.1 m above ground, but then abundance rapidly decreases to values between ∼16% and ∼15.5% at 0.5 m and 1.0 m, respectively.

Figure 9 represents the same plots shown in Fig. 8 but for DD #2. The distribution of clay, silt, and sand is shown in Fig. 9a. Within the first 0.25 m of sampling, clay and silt increase from ∼11.5% and ∼18% to ∼24.5% and ∼31.5%, respectively. Sand shows a contrary behavior at the same heights, decreasing from ∼39% abundance to ∼29%. Between 0.75 and 1 m, all size categories decrease to a similar abundance of ∼12%. In the upper parts of the dust devil, the trends differ for each size category. Clay shows an irregular trend but maintains a relatively high abundance of ∼15.5% at 2 m height. Silt content generally decreases slightly above 0.75 m height. Sand decreases rapidly in abundance with height, up to 1 m height, and then shows a slower, more constant decrease above 1 m. The detailed distribution of clay grain sizes is presented in Fig. 9b. No clear trend is visible in either clay size categories, however, although both show a peak in abundance at 0.75 m height. The detailed grain size distribution of silt is presented in Fig. 9c. It is obvious that very fine silt, fine silt, and medium silt increase in abundance between 0.50 and 0.75 m, but coarse silt does not. This peak is more distinct for the finer materials. Between 0.75 and 1 m height, all silt sizes decrease by different proportions: finer-grained material decreases in abundance more rapidly than larger-grained material (*e.g.,* very fine silt decreases in abundance from ∼39.5% to ∼10%, while coarse silt decreases only from ∼22% to ∼15.5%). Above 1 m, all silt grain sizes show a decrease in abundance with height, with only some small variations. The detailed grain size distribution for sand is shown in Fig. 9d. The general trend of all grain size categories shows a rapid decrease in abundance with height at low height, followed by a slowly decreasing or steady abundance higher in the dust devil. Medium sand decreases especially rapidly in the first 0.5 m above ground: from ∼71.5% abundance at 0.5 m to ∼2% abundance at 1 m height. Absolute values for the data shown in Figs. 8 and 9 are provided in Table 3.

**Table 3.** Grain Size Distribution for Every Sample Height of Both Dust Devils Presented in Figures 8 and 9

	*DD #1*								
*Height (m)*	*Clay <2 μm*	*Clay 2 to <4 μm*	*Very fine silt 4 to <8 μm*	*Fine silt 8 to <16 μm*	*Medium silt 16 to <31 μm*	*Coarse silt 31 to <63 μm*	*Very fine sand 63 to <125 μm*	*Fine sand 125 to <250 μm*	*Medium sand 250 to <500 μm*
0.1	341	276	218	169	159	182	560	934	127
0.5	257	250	161	149	111	111	425	675	41
1.0	128	134	159	110	79	75	228	387	39
1.5	141	247	196	103	64	54	189	313	14
2.0	111	241	173	113	76	52	127	167	10
2.5	247	362	229	173	119	80	106	170	7
3.0	215	351	198	125	81	57	119	119	5
3.5	169	401	230	172	120	70	81	83	7
4.0	186	306	175	109	65	41	65	68	2
**Total**	**1609**	**2262**	**1564**	**1114**	**809**	**681**	**1835**	**2848**	**250**

	*DD #2*								
*Height (m)*	*Clay <2 μm*	*Clay 2 to <4 μm*	*Very fine silt 4 to <8 μm*	*Fine silt 8 to <16 μm*	*Medium silt 16 to <31 μm*	*Coarse silt 31 to <63 μm*	*Very fine sand 63 to <125 μm*	*Fine sand 125 to <250 μm*	*Medium sand 250 to <500 μm*
0.5	158	210	182	167	198	160	253	385	40
0.75	286	492	514	365	225	130	213	275	11
1.0	205	181	131	153	140	92	107	94	1
1.25	211	268	138	126	129	82	71	54	3
1.5	217	135	129	126	92	48	69	45	0
1.75	192	115	91	87	74	33	40	19	1
2.0	313	176	111	105	61	44	33	15	0
**Total**	**1269**	**1401**	**1185**	**1024**	**858**	**545**	**753**	**872**	**56**

## 5. Discussion

### 5.1. Vertical grain size distribution

Both dust devils investigated in this study show similar trends in their grain size distribution within the dust devil (aside from clay, which shows an increase of grains with size in DD #1 and relatively constant values for DD #2) and in their lifted particle load versus height (nearly exponential decrease). In both dust devils, fine sand (125 to <250 μm) contributes about 50 wt % to the total mass of lifted particles. This is most likely affected by the composition of the surface, in which fine sand has a contribution of ∼33.7 wt % and is the second largest fraction after very coarse sand (∼35.4 wt %). We measured only one “very coarse sand” grain for each sample, which is under 0.01% of all lifted particles, so we assume that this grade of sediment was not usually lifted by these dust devils. Also, the values of contributions to the total mass of the other grain sizes show comparable amounts in both dust devils. This is probably due to the same soil grain size distribution from which both dust devils eroded material, indicating that dust devils somewhat represent the surface they move over. Furthermore, the trends (and not the values themselves) of the calculated mean values and medians of the diameters of grains in the different sample heights are comparable. This is interesting in that both dust devils had different sizes and intensities, and the sample heights and the sample intervals were different. These observations imply a similar or comparable internal structure of both dust devils, despite their different strengths and dimensions.

Using laboratory simulations of dust devils' dust flux, Neakrase *et al.* (2006) and Neakrase and Greeley (2010) concluded that the strength of vortices that lift sedimentary loads is not directly linked to their size but to their strength of the pressure drops in their core. This was indirectly confirmed by our measurement due to the comparable internal structures of the two different dust devils.

In a comparison of *in situ* sampling and grain size distribution measurements within the vortex of dust devils, Oke *et al.* (2007) showed some striking similarities. In Australia, Oke *et al.* (2007) performed *in situ* measurements by taking samples of 20 active dust devils at heights up to 1.6 m with a sample interval of 0.1 m. They measured silt- and sand-sized material and classified them in four different groups as follows: medium silt (6–20 μm diameter), coarse silt (20–63 μm diameter), fine sand (63–200 μm diameter), and medium sand (200–600 μm diameter). Their results indicate that approximately 80% of the total number of lifted grains by a dust devil were smaller than 63 μm. This is in agreement with our measurements of DD #2 where ∼80.5% of all lifted particles were smaller than 63 μm. In DD #1, same measurements show values of ∼64%, which are smaller but comparable to the results of Oke *et al.* (2007).

Oke *et al.* (2007) stated that less than 1% of the collected particles were medium sand grains (200–600 μm), which again is in good agreement with our results [medium sand (250 to <500 μm): ∼1.8% for DD #1 and ∼0.6% for DD #2]. In contrast, Mattsson *et al.* (1993) analyzed grain samples from a dust devil in southern Tunisia without presenting the height at which they collected the samples. Their measurements show a composition of about 42% fine sand and ∼58% silt and clay for the dust devil, though they did not specify the diameters of the grain size categories (Mattsson *et al.,* 1993). After the classification of Udden (1914), our results show that (by particle number) ∼31.2% of clay, ∼32.6% of silt, and ∼36.6% of sand were lifted in DD #1 and ∼35.8% of clay, ∼44.6% of silt, and ∼19.6% of sand were lifted in DD #2. This is a generally lower amount of lifted sand compared to the work of Mattsson *et al.* (1993).

The results of this study show that the majority of large grain sizes (sand) were only lifted within the first meter of sampling. In DD #1, ∼69% of all lifted sand grains (very fine sand to medium sand) and, in DD #2, ∼82% of all lifted sand grains were sampled within the first meter of the vortices. In total, ∼46.5% (for DD #1) and ∼61% (for DD #2) of all lifted grains were lifted only within the first meter. This was also described by Oke *et al.* (2007) and Metzger *et al.* (2011), who observed that large grain sizes were only lifted within the first decimeters above ground. Our results also show that ∼76.5 wt % (DD #1) and ∼89 wt % (DD #2) of the total particle load of the measured dust devils were lifted within the first meter of the vortices. This is in good agreement with the results of Metzger *et al.* (2011), who suggested that ∼85% to 95% of the dust devils' basal sediment load is coarse-grained. All these observations are direct evidence for the existence of a sand skirt. This sand skirt contains the majority of lifted material, but the large grain sizes are not entrained into the atmosphere, in that they fall promptly back to the surface (Greeley *et al.,* 2004, 2006; Balme and Greeley, 2006; Whelley and Greeley, 2008; Reiss *et al.,* 2013). A sand skirt is normally wider than the vortex, and larger particles can continue their movement over the surface in saltation after they fall back onto the surface (Greeley *et al.,* 2006). A general trend of a decrease in grain sizes and, therefore, lifted particle load with height is clearly discernible in both sample sets.

### 5.2. Particle load

Metzger *et al.* (2011) measured the dust loads of dust devils but generally disregarded the vertical grain size distribution of the lifted material. They concluded that ∼10 wt % of the total lifted material contained grains between 0.1 and 10 μm, which can be transported over large distances within the atmosphere, while the remaining ∼90 wt % was only transported close above the surface in the sand skirt (Metzger *et al.,* 2011). Our results show different, much lower values. If we assume that grains with a diameter <25 μm could get into suspension (Gillette and Sinclair, 1990; Balme and Greeley, 2006) and we expand this grain size up to <31 μm (clay to medium silt) to fit with our measurements, we still have a very low portion of only ∼0.05 wt % lifted particle load for DD #1 and ∼0.15 wt % for DD #2. Although we have expanded the grain size to a factor of 3 compared to that of Metzger *et al.* (2011) (<2 to <31 μm in contrast to 0.1–10 μm), the difference is large.

We propose several explanations for such significant differences, as follows: (i) the measured dust devils could have been different in terms of their dimensions and strengths in the two study areas (North America and Africa), or (ii) there could have been different surface compositions, possibly with more fine-grained minerals in the study region investigated by Metzger *et al.* (2011). We believe the second explanation probably contributes most to the difference in results; the dust devils in our study were sampled on a sandy desert surface, while Metzger *et al.* (2011) sampled dust devils mostly on playa surfaces with zones of fine and coarse surficial material.

As mentioned before, dust devils can have an influence on the atmospheric dust load by the transport of fine-grained material into the atmospheric boundary layer. Although our measurements have shown that only ∼0.05 to 0.15 wt % of the lifted particle load can go into suspension, the number of relative particles that can go into suspension (in our measurements grain sizes up to <31 μm) is much higher (∼58.5% for DD #1 and ∼73.5% for DD #2). These measurements were only made within the first 2 and 4 m, respectively (the columns of both sampled dust devils were much higher); nevertheless, it shows that the relative contribution of fine-grained particles within the first meters of a dust devil vortex is also high. Our measurements also show that the relative amount of small particles (clay and very fine to medium silt) increases with height.

The composition of the dust devil vortex could also be affected by the surface composition over which dust devils move. In our study region, the second largest fraction of grain sizes of the surface are fine sand grains with ∼33.7 wt % (the largest fraction are very coarse sand grains, but they were not extensively lifted). These grain sizes also represent the largest fraction of grains sampled within both dust devils (∼54.9 wt % for DD #1 and ∼48.8 wt % for DD #2).

Each day in the field, we observed several larger dust devils outside our study region that were up to several hundred meters tall and had diameters of several tens of meters. Although these dust devils could not be sampled, their visibility from afar and their dimensions imply a much higher input of fine-grained material into the atmosphere than the relatively small dust devils that were sampled in this study. It remains to be measured whether there is a much higher abundance of fine-grained material in larger, more intense dust devils.

### 5.3. Dust devil tracks

Dust devil tracks within the study area were detected in satellite imagery. Also, during the field campaign, several weak, dark surface tracks left by dust devils were observed (Reiss *et al.,* 2012). Furthermore, the surface of the study area shares comparable characteristics with the surface of the desert region in western China where dust devil tracks on Earth were first investigated *in situ* (Reiss *et al.,* 2010, 2011a). Despite these observations, neither of the analyzed dust devils in this survey left visible tracks. The reason for this behavior is unclear, but there are different possible reasons, as follows: (i) Reiss *et al.* (2010) stated that a dust layer on top of coarser-grained material is required for the formation of dust devil tracks, so a lower availability of fine-grained material (dust) on the surface may be a factor. This could indicate that the present study area is not as dusty as other regions on Earth where dust devil tracks are more abundant, or simply that the dust availability at the time of our *in situ* sampling was low. (ii) It is also possible that the measured dust devils were too weak to erode enough dust material from the surface to engender a visible track.

Another possible mechanism for the formation of dust devil tracks is the redistribution of sand-grade material (>500 to 2000 μm) that would lead to the formation of cycloidal-patterned tracks (Reiss *et al.,* 2013). Although sand-sized material was directly measured in our dust devil, especially in near-surface heights, the amount of sampled coarse sand grains (>500 μm) in both dust devils was negligible, which indicates that neither dust devil in our study was strong enough to redistribute large amounts of coarse sand grains.

## 6. Conclusions

This study presents detailed grain size distributions and relative lifted particle loads for two dust devils up to 4 m high. The results provide direct measurement of larger materials that are constrained to the lower parts of the dust devils: ∼69% to ∼82% of all lifted sand-grade grains were transported only within the first meter of the vortices. Furthermore, the lowest regions contain ∼76.5 wt % (DD #1) and ∼89 wt % (DD #2) of the total lifted particle load. Hence, this provides verification of the existence of sand skirts in both dust devils.

Our measurements show that ∼58.5% and ∼73.5% of the number of lifted particles could possibly go into suspension, but this fraction only represents ∼0.05 to 0.15 wt % of lifted particle load. Also, we confirmed a vertical trend of decreasing particle size with height within dust devils that leads to a nearly exponential decrease of particle load with height.

The comparable trends of the vertical grain size distributions and the relative lifted particle load in the two measured dust devils indicate a comparable internal particle load structure for the dust devils, despite their different dimensions and strengths.
